# Community Structure and Ecological Network's Changes of Vaginal Microbiome in Women Right After Delivery

**DOI:** 10.3389/fped.2022.750860

**Published:** 2022-03-28

**Authors:** Hongping Li, Jingbo Jiang, Chuan Nie, Bin Xiao, Qingxia Li, Jieyang Yu

**Affiliations:** ^1^Department of Neonatology, Shenzhen Children's Hospital, Shenzhen, China; ^2^Department of Neonatology, Guangdong Women and Children Hospital, Guangzhou, China; ^3^Department of Critical Care Medicine, Baise Maternal and Child Hospital, Baise, China

**Keywords:** vaginal microbiota, neonatal oral microbiome, community state type, 16S rRNA sequencing, ecological network

## Abstract

**Objectives:**

Vaginal microbiota is not only an important source of bacterial colonization for neonates, but also plays a crucial role in maternal and neonatal health. This study aimed to investigate the vaginal microbial community structure right after delivery and its impact on the neonatal oral microbiome.

**Methods:**

In this study, 27 women were recruited from Bao'an Maternal and Child Health Hospital. Bacterial compositions of vaginal samples before and right after delivery and neonatal oral samples right after birth were investigated using 16S rRNA sequencing of V3–V4 hyperregions.

**Results:**

Vaginal microbiome before delivery was dominated by the genus *Lactobacillus*. After delivery, the vaginal microbial community was altered, with significantly decreased proportion of *Lactobacillus*, increased alpha-diversity, and a more diverse ecological network. A large number subjects dominated by *Lactobacillus* species before delivery shifted to CST (community state type) IV after delivery. In addition, similar changes were observed in the neonatal oral microbiome, and its community profile was closer to vaginal samples after delivery than before delivery with principal coordinates analysis and microbial source tracking analysis.

**Conclusion:**

The vaginal microbiome was altered right after delivery and impacted the colonization of the neonatal oral microbiome in China. Further, it is vital to understand the longitudinal influence on maternal and neonatal health of vaginal microbiome community changes after delivery.

## Introduction

The vaginal microbiome is dynamic and influenced by many physiological changes such as sexual development, sexual intercourse, menstrual cycle, pregnancy, menopause, and hormone levels (1). Accumulating evidence proves that resident vaginal microbiota is related to bacterial vaginosis, menstruation, and sexually transmitted infections through interfering the proliferation of some organisms (1–4). Notably, dysbiosis of the vaginal microbiome community during pregnancy is associated with an increased risk of postpartum endometritis, preterm delivery, and spontaneous abortion (5–7). Not only as an important factor for women's reproductive health, vaginal microbiome in pregnancy also plays a key role in neonatal health (8). It is also understood that the bacterial community of the vagina of pregnant women is typically dominated by *Lactobacillus* species (9), many of which can produce biosurfactants and bacteriocins antagonistic to pathogens (10).

Understanding the community structure of the vaginal microbiome in pregnancy is important for maternal and neonatal health, which draws more and more attention. Several cross-sectional and longitudinal cohorts have been examined in studying the vaginal microbiome during pregnancy (9, 11, 12). Collectively, the results of these studies suggest that pregnancy leads to great stability, increases *Lactobacillus* proportional abundance, and reduces the richness and diversity of vaginal microbiome, although only a few studies investigate postpartum vaginal microbiome (13, 14). During the postpartum period, the vaginal microbiome is characterized by fewer *Lactobacillus* species and more diverse than that of pregnancy phase. However, all the vaginal samples included in the postpartum period are a few weeks after delivery; a comprehensive characterization of vaginal microbiome signature right after delivery has not yet been undertaken. The aim of this study was to characterize the composition of the vaginal microbiome right after delivery.

Neonates have been exposed to the maternal vaginal microbial ecosystem during the delivery process, and microbiota colonized at the vaginal birth canal is introduced to the neonate (15, 16). These pioneer microbial colonizers of the neonate, especially the gut and oral microbiome, play critical roles in neonatal health and development, including nutrient acquisition, immune programming, and protection from pathogens (16, 17), as the neonatal oral microbial structure is affected by multiple environmental factors, such as the maternal vaginal canal, skin-to-skin contact, maternal breast milk feeding, and so on (18, 19). As a valuable source of pioneer bacteria for neonatal oral microbiome, it is important to discern the relative potential contribution of the maternal vaginal community to the neonate oral microbiome. Herein, we tried to investigate the associations between the neonatal oral microbiome and vaginal microbiome before and right after delivery, respectively.

## Methods

### Study Subjects

Twenty-seven healthy and reproductive-aged women with gestational age >37 weeks were recruited in this study. They were asymptomatic and showed no clinical signs of vaginal disease upon examination by obstetricians. They were also with an uncomplicated singleton pregnancy and without medical problems or adverse outcomes during pregnancy, without known fetal anomalies or complications, and without antibiotics or other antimicrobial therapy during pregnancy. This study received ethics approval from Bao'an Maternal and Child Health Hospital (Shenzhen, China, no. LL2020012104). Written informed consent was obtained from all participants and parents/guardians of the recruited newborns. After obtaining written informed consent, all participants' demographic and clinical characteristics were collected via interview and by reviewing medical charts, including gestational age, height, weight, blood pressure, body mass index (BMI), ethnicity, age, and so on. Relevant clinical information was also obtained from neonates at birth.

Vaginal samples before delivery were collected at the first examination of hospital admissions of all participants (group BD, before delivery). Vaginal samples after delivery were collected before mothers left the delivery room with incision stitched (group AD, after delivery). The sterile swab was placed carefully on the vaginal sidewall about halfway between the introitus and the cervix, following the instructions reported previously (12), pressed firmly into the sidewall to a depth of roughly the diameter of the swab, rolled dorsally–ventrally back and forth four times to coat the swab. Neonatal oral samples were taken as soon as the newborns were delivered and before feeding by carefully swabbing the oral mucosa (group NO, neonatal oral) according to the previous study (20). Three sterile swabs were obtained for every sample by trained nurses to avoid insufficient DNA concentration. All samples were stored at 4°C and transferred to −80°C storage within 30 min after collection until DNA extraction.

### Microbiome Profiling

DNA was extracted from vaginal and oral swabs using QIAamp DNA Mini kit (Qiagen, Germany). The concentrations and purity were measured using the NanoDrop One (Thermo Fisher Scientific, Waltham, MA, USA). The average DNA concentration was 41.52 ng/μL per sample. The DNA concentration of vaginal samples before delivery (69.68 ± 85.19 ng/μL) was higher than the samples after delivery (33.15 ± 49.63 ng/μL) and higher than that of the neonatal samples (21.71 ± 45.50 ng/μL).

V3–V4 hypervariable regions of 16S rRNA genes were amplified with forward primer 338F (5′-ACTCCTACGGGAGGCAGCAG-3′), reverse primer 806R (5′-GGACTACHVGGGTWTCTAAT-3′). Polymerase chain reaction (PCR) reactions, containing 25 μL 2 × Premix Taq (Takara Biotechnology, Dalian Co. Ltd., China), 2 μL of each 10 mM primer and 3 μL DNA template in a volume of 50 μL, were amplified under the following thermal profile: 94°C for 5 min, then 30 cycles of 94°C for 30 s, 52°C for 30 s, 72°C for 30 s, followed by 72°C for 10 min. Amplification products were visualized with 1% agarose gel electrophoresis. These PCR products were then pooled in equimolar and paired-end sequences on an Illumina MiSeq machine (Illumina, Inc., San Diego, CA, USA) with V3 chemistry.

Sequencing reads were assigned to each sample according to barcode sequences and filtered reads containing ambiguous bases or mismatches in the primer regions using custom Perl scripts. Then, the 16S rRNA gene sequences generated were analyzed using the bioinformatics software package QIIME2 (version 2019.4) (21). Paired-end reads were first denoised via DADA2 (22) offered by QIIME2 with command “qiime dada2 denoise-paired,” to merge paired-end reads and quality filtering and to exclude chimeric and phiX sequences. Further sequences were classified against Greengenes (13_8 revision) database at the species level using command “qiime feature-classifier classify-sklearn.” Meanwhile, an array of alpha- and beta-diversity measures was generated using command “qiime phylogeny align-to-tree-mafft-fasttree” and “qiime diversity core-metrics-phylogenetic” at a sampling depth of 1,000. Alpha-diversity was calculated by Shannon's diversity index, observed feature number, and Pielou's measure of species evenness. Weighted and unweighted UniFrac distances were used to represent beta-diversity.

Moreover, sequences were separately analyzed using Mothur software against Greegenes (13_8 revision) database as described previously (20, 23), where the sequences were clustered into OTUs (operational taxonomic units) based on the similarity threshold of 0.97.

### Statistical Analysis

The continuous demographic variables were presented as the mean ± standard deviation (SD), including alpha- and beta-diversity indexes. The categorical characteristics were reported as numbers (%). All comparisons of this study were performed in R software at 0.05 level of significance using χ^2^ and *t*-tests for categorical and continuous variables, respectively.

Principal coordinates analysis (PCoA) was performed based on unweighted and weighted UniFrac distances using QIIME2 software and accompanied with permutational multivariate analysis of variance (PERMANOVA, 999 permutations) with R package “vegan.” LEfSe (linear discriminant analysis effect size) software (24) was used to analyze the significant different microbes between two groups with linear discriminant analysis >3.0 and *P* < 0.05. The microbial ecological network was constructed using the SPIEC-EASI method (25) and visualized with Gephi 0.9.2 software. SourceTracker 1.0.1 software (26) was further used to investigate the transmission from vaginal microbiome to neonatal oral microbiome.

Assignments of vaginal microbial profiles to community state types (CSTs) were performed according to community composition, and the community clustering was performed using McQuitty linkage hierarchical clustering analysis with R software as previously reported (12).

## Results

### Maternal and Neonatal Clinical Data

Demographic and clinical characteristics of the women and newborns are provided in Table 1. A total of 27 healthy and asymptomatic women were recruited into this study. All of the subjects were Han ethnicity ranging from 19 to 34 years old (mean age, 28.1 years). The average BMI was 25.2 kg/m^2^ (range, 21.1–34.1 kg/m^2^). All women gave birth between the 37th and 42nd gestational week with 14.26-kg (range, 9.0–26.30 kg) average gestational weight gain. The average birth weight of 27 newborns was 3,205.9 g (range, 2,600–3,850 g), including 15 boys and 12 girls.

**Table 1:** Demographic and clinical data for study participants.

**Characteristics**	**Value**
**Maternal conditions**	
Mother's age, years, mean (SD)	28.1 ± 3.3
Mother's BMI, kg, mean (SD)	25.2 ± 2.6
Gestational weight gain, kg, mean (SD)	14.26 ± 0.37
Gestational week, mean (SD)	39.5 ± 1.0
Hospital stay before labor, hours, mean (SD)	16.8 ± 14.8
First degree of perineal laceration, *n* (%)	18 (66.7%)
**Neonatal conditions**	
Children sex (male), *n* (%)	15 (55.6%)
Birth weight, g, mean (SD)	3,205.9 ± 289.2
Apgar score, mean	10

### Changed Microbial Community After Delivery Accompanied With High Alpha-Diversity

**Figure 1:**
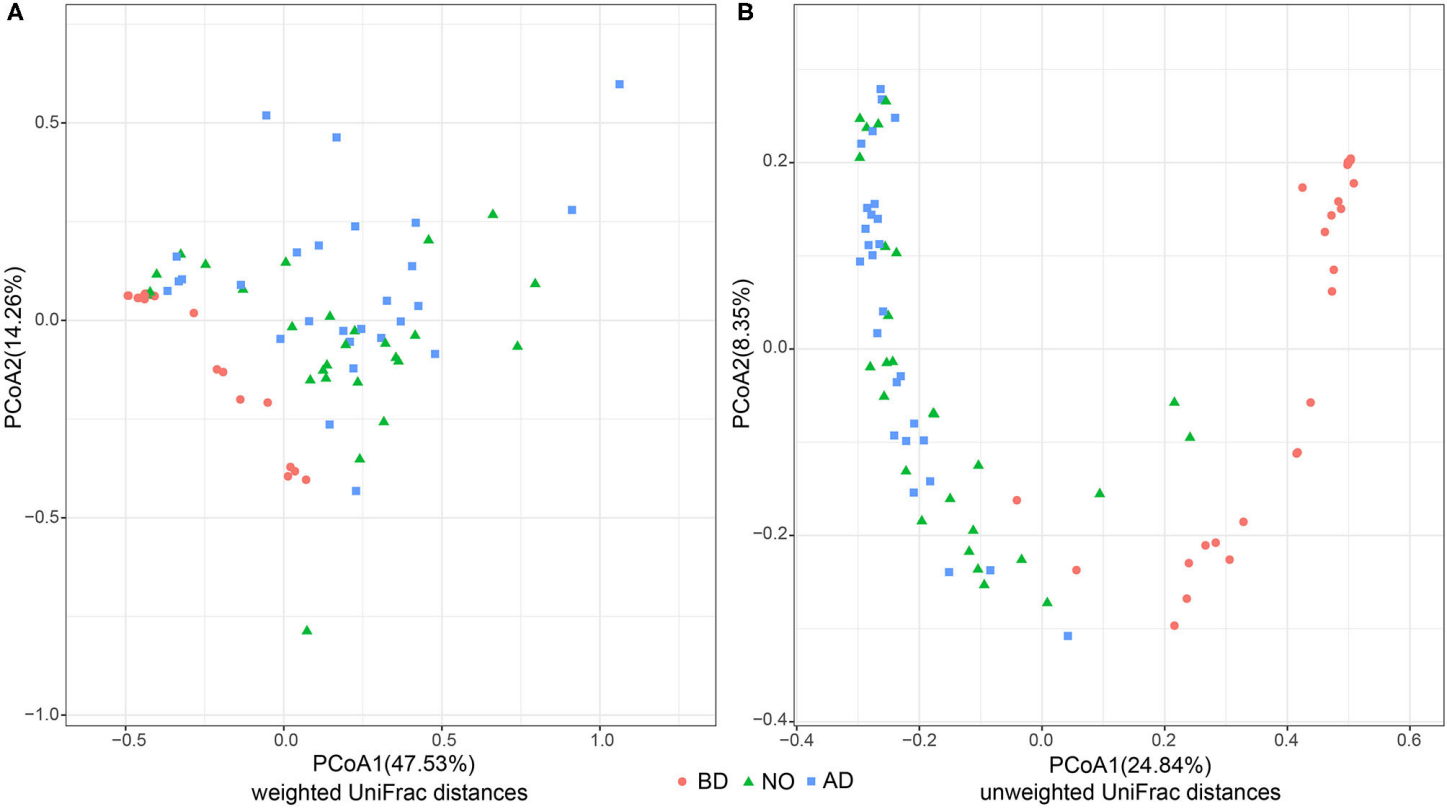
PCoA analysis of three groups based on weighted and unweighted UniFrac distances (group BD, before delivery; group NO, neonatal oral; group AD, after delivery). **(A)** PCoA analysis performed on weighted UniFrac distances. **(B)** PCoA analysis performed on unweighted UniFrac distances.

To compare the overall vaginal community before and after delivery, PCoA was implemented on weighted and unweighted UniFrac distances separately (Figure 1). Both weighted and unweighted UniFrac distances showed that vaginal samples before delivery separated well from the subjects after delivery with PERMANOVA (*R*^2^ = 0.281, *P* = 0.001; *R*^2^ = 0.283, *P* = 0.001). Significant differences were also observed between vaginal samples before delivery and neonatal oral samples in unweighted (*R*^2^ = 0. 229, *P* = 0.001) and weighted UniFrac distances (*R*^2^ = 0. 261, *P* = 0.001), whereas a subtle difference was observed in unweighted UniFrac distances when comparing vaginal samples after delivery and neonatal oral samples (*R*^2^ = 0.027, *P* = 0.038), but no difference in weighted UniFrac distances (*R*^2^ = 0.026, *P* = 0.172). It indicated that the neonatal oral microbial community was closer to the vaginal microbial community after delivery than before delivery. Further, SourceTracker analysis demonstrated that an average of 30.11% neonatal oral microbiota potentially transmitted from vaginal microbiome before delivery, whereas 79.11% potentially transmitted from vaginal microbiome after delivery.

**Figure 2:**
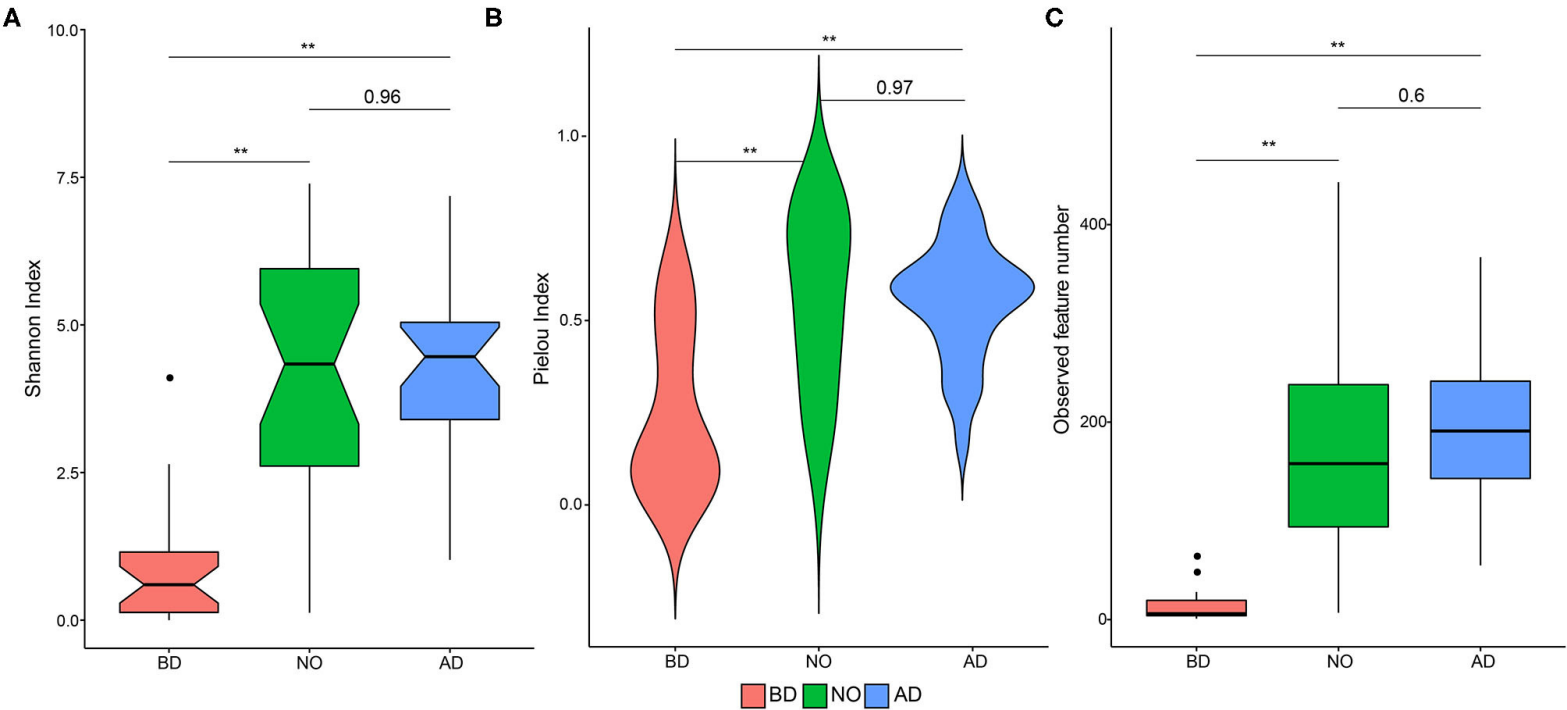
Alpha-diversity plots in microbial community and the comparisons among the three groups (group BD, before delivery; group NO, neonatal oral; group AD, after delivery). **(A)** Shannon index, **(B)** Pielou index, **(C)** observed feature number. Notes, **means *P* value < 0.01.

The vaginal microbiota after delivery and neonatal oral microbiota were associated with higher alpha-diversity, evenness, and richness (Figure 2). The mean Shannon index value was 0.92 ± 1.06 of the BD group, which was significantly lower than that of group AD (4.22 ± 1.50, *P* < 0.01) and NO (4.19 ± 2.22, *P* < 0.01) (Figure 2A). Increased mean Pielou index values were also observed in the AD (0.55 ± 0.16, *P* < 0.01) and NO (0.56 ± 0.24, *P* < 0.01) groups compared with group BD (0.25 ± 0.22) (Figure 2B). Meanwhile, the mean observed feature number increased significantly in neonatal oral samples (179.89 ± 113.37 vs. 13.30 ± 14.83, *P* < 0.01) and in vaginal samples after delivery (194.33 ± 85.69 vs. 13.30 ± 14.83, *P* < 0.01) (Figure 2C), compared with the vaginal samples before delivery.

### Compositional Differences of Vaginal Microbiome Between Before and After Delivery

**Figure 3:**
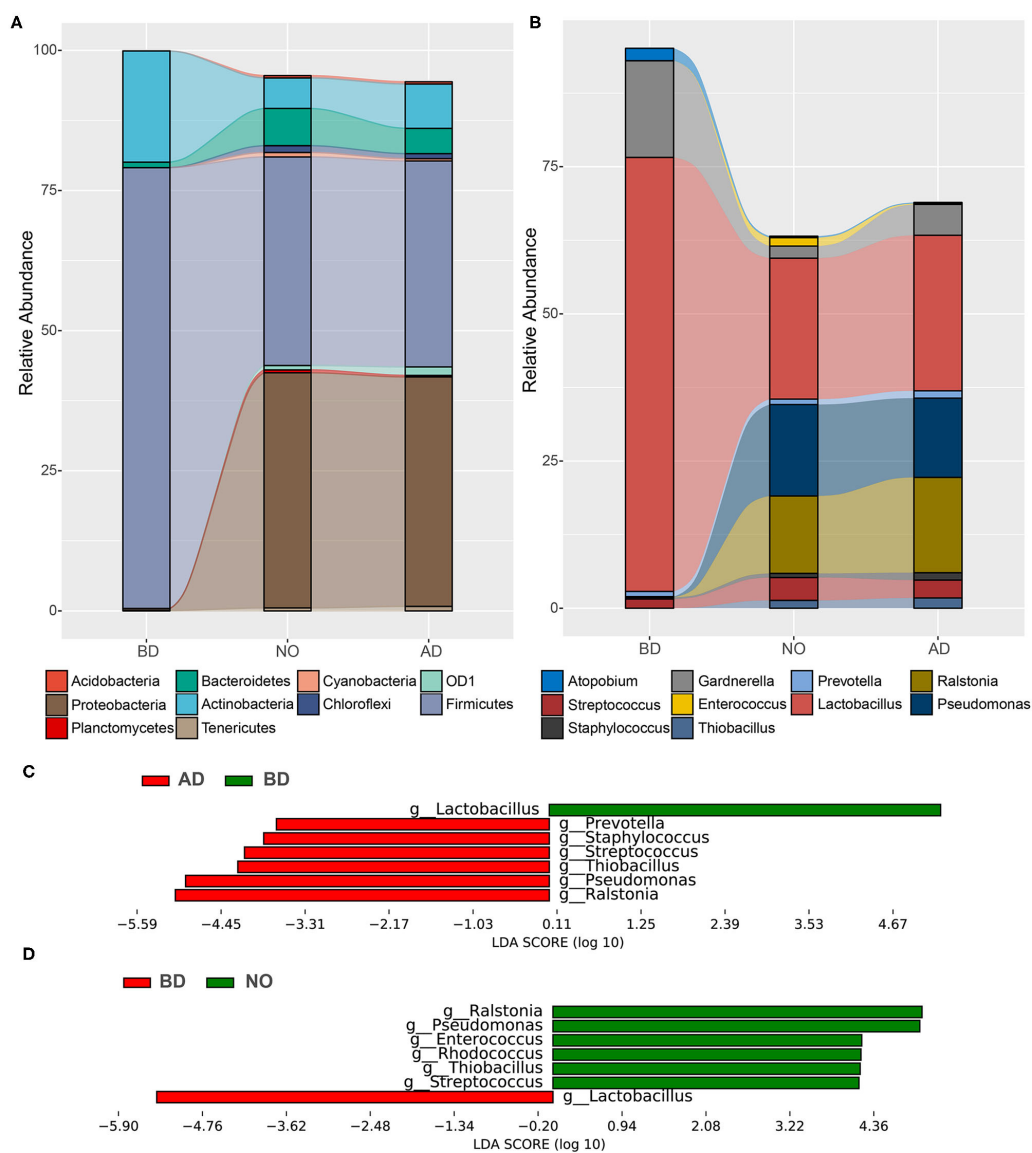
Relative proportions and differences of abundant microbes among the three groups. **(A)** Relative abundance at the phylum level. **(B)** Relative abundance at the genus level. **(C)** Significant different genera between BD and AD groups. **(D)** Significant different genera between the BD and NO groups.

The microbiome of study participants were characterized using high-throughput sequencing of the 16S rRNA V3–V4 high-variable regions. A total of 3,788,402 reads were included in the analysis. The average sequence read count was 46,770 per sample, with a median of 46,158 (range, 13,434–71,182), and the mean and median read lengths were 420 and 423 base pairs, respectively. The average relative abundance of each group at the phylum and genus levels is shown in Figure 3. It showed that the top 10 phyla were *Acidobacteria, Actinobacteria, Bacteroidetes, Chloroflexi, Cyanobacteria, Firmicutes, OD1, Planctomycetes, Proteobacteria*, and *Tenericutes* (Figure 3A). Meanwhile, the top 10 genera were *Atopobium, Enterococcus, Gardnerella, Lactobacillus, Prevotella, Pseudomonas, Ralstonia, Staphylococcus, Streptococcus*, and *Thiobacillus* (Figure 3B).

The results also demonstrated that pregnancy was associated with a microbiome largely dominated by phyla *Firmicutes* (average, 78.65%) and *Actinobacteria* (average, 19.83%), which accounted for more than 98% of the microbial population, while substantial shifts in bacterial phylum community were observed at neonatal mucous and vaginal samples after delivery (Figure 3A), with dramatically decreased proportions of *Firmicutes* (37.20 and 36.75%) and *Actinobacteria* (5.44 and 7.92%), increased abundances of *Proteobacteria* (41.94% and 40.93% vs. 0.35%) and *Bacteroidetes* (6.64% and 4.52% vs. 0.97%). As the important genus in phylum *Firmicutes*, the average proportion of *Lactobacillus* decreased from 73.71% before delivery to 26.42% after delivery and 23.94% in neonatal oral, accompanied with increases in genera *Streptococcus* (3.04% vs. 1.56%), *Ralstonia* (16.19% vs. 0.04%), *Pseudomonas* (13.45% vs. 0.09%), and *Thiobacillus* (1.74% vs. 0%) (Figure 3B).

Next, the LEfSe software was used to detect significant differences in relative abundances of genera, accounting for >1% on average in at least one group. Figure 3C included a list of genera that are significantly different between vaginal samples before and after delivery. Among them, the genera *Prevotella, Staphylococcus, Streptococcus, Thiobacillus, Pseudomonas*, and *Ralstonia* increased significantly after delivery, whereas genus *Lactobacillus* decreased significantly. In addition, the significantly different genera between vaginal samples before delivery and neonatal oral samples are presented in Figure 3D. Compared with vaginal samples before delivery, the relative abundances of genera *Ralstonia, Pseudomonas, Enterococcus, Rhodococcus, Thiobacillus*, and *Streptococcus* were higher, whereas genus *Lactobacillus* was dramatically lower in neonatal oral samples. Notably, there was no difference between vaginal samples after delivery and neonatal oral samples. These observations collectively suggested that the vaginal microbial composition after delivery was significantly different from before delivery.

### Different Ecological Network of Vaginal Microbiome Between Before and After Delivery

**Figure 4:**
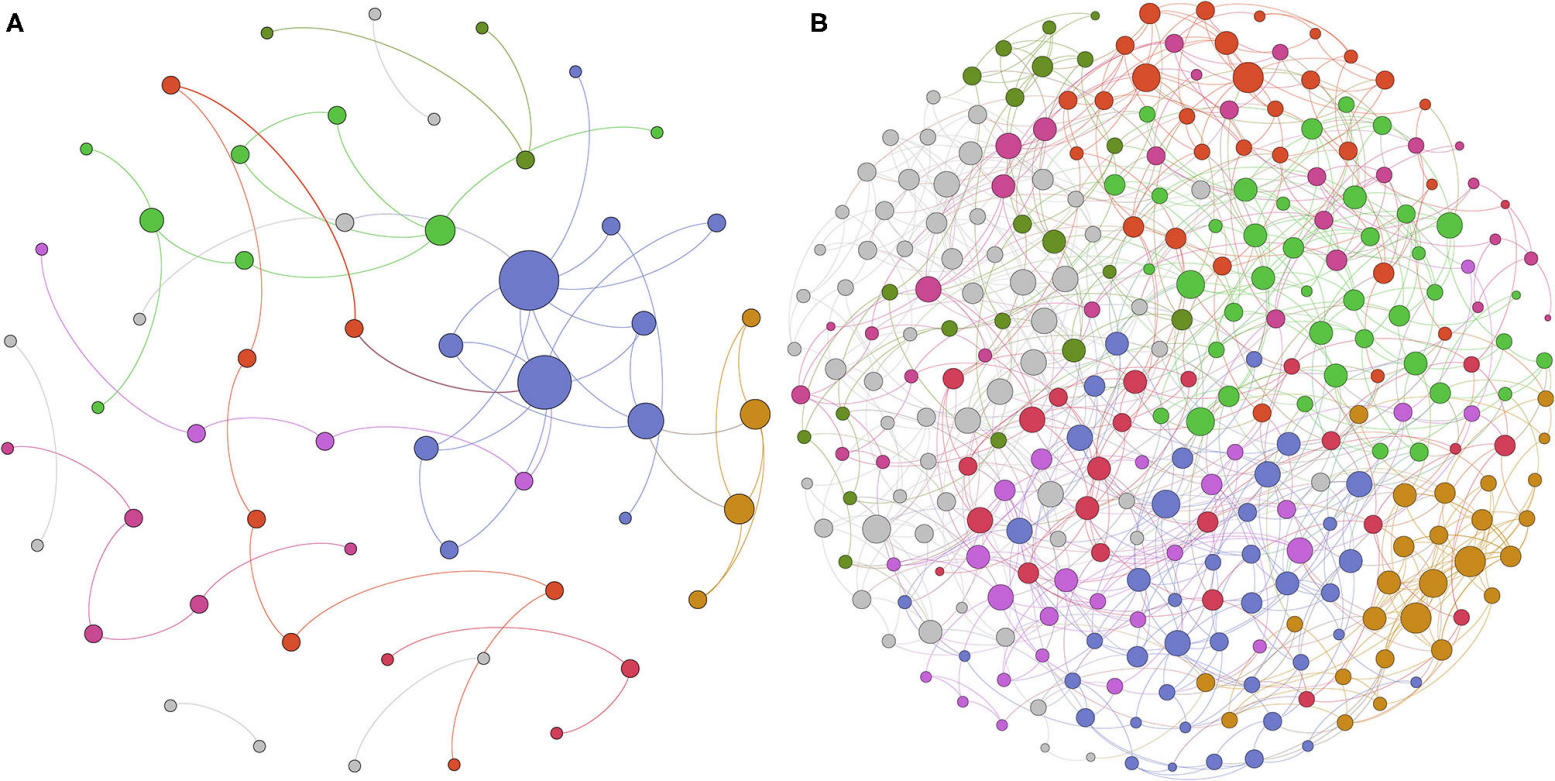
Different microbioal ecological network between before and after delivery with SpiecEasi analysis. **(A)** Microbial ecological network at the family level in the BD group. **(B)** Microbial ecological network at the family level in the AD group.

SpiecEasi software was used to construct ecological association networks at the family level of the vaginal microbiome before and after delivery, respectively. The results demonstrated that the ecological network of group BD included 55 nodes and 59 edges (Figure 4A). Modularity analysis indicated that family Lactobacillaceae coexisted with Bifidobacteriaceae. However, 293 nodes and 850 edges were presented in the AD group (Figure 4B), and the family Lactobacillaceae coexisted with other 40 families with modularity analysis. This was in accordance with the results observed by PCoA, indicating an obviously different vaginal microbial community right after delivery.

### Community State Types Analysis and Its Changes After Delivery

**Figure 5:**
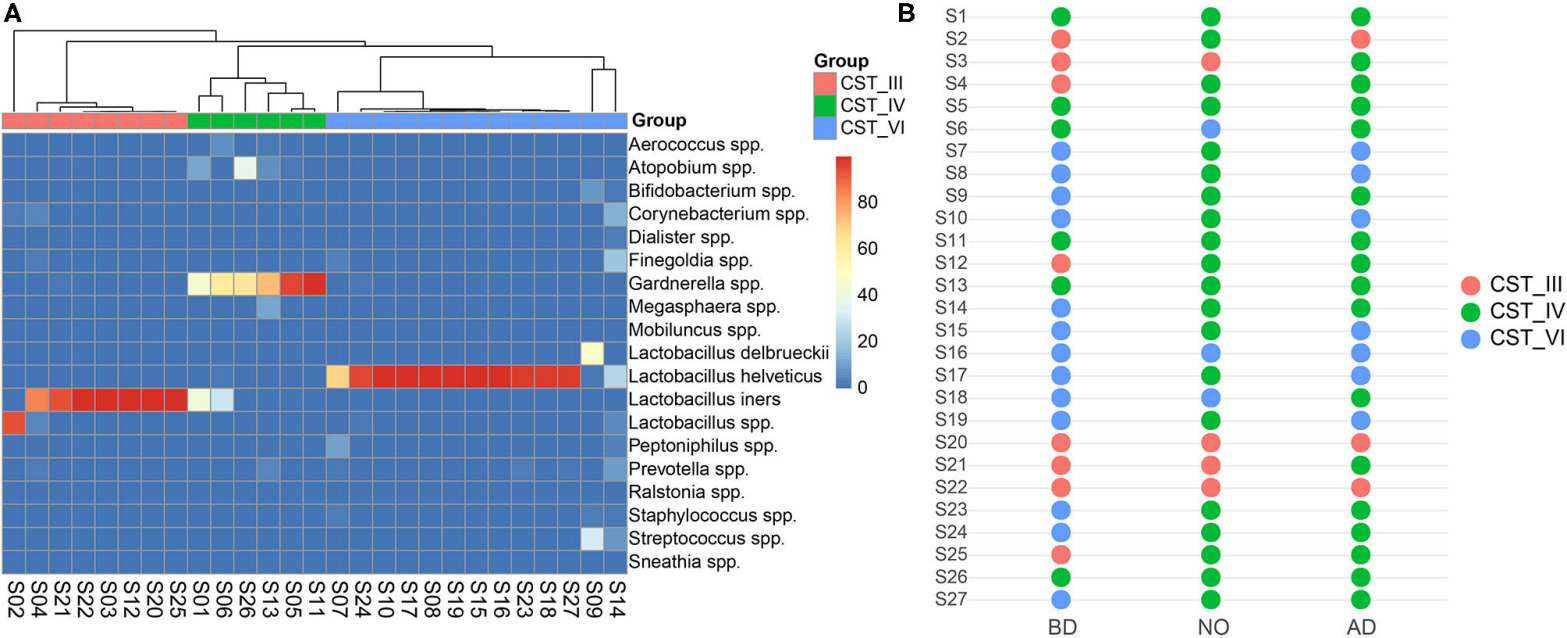
Vaginal community state types presented throughout before and after delivery. **(A)** Hierarchical clustering analysis using McQuitty linkage of microbial species abundance showed that these subjects were clustered into three major groups. **(B)** The switch of community state types among the three groups.

Categorizing microbiome profiles based on the taxon with the largest proportion of reads and hierarchical clustering analysis of bacterial species from the pregnant vaginal microbiome profiles revealed three major CSTs (Figure 5A). Among all samples, eight samples were dominated by the species *Lactobacillus iners* (CST III). Six samples were assigned to CST IV, which were dominated by the genus *Gardnerella*, and also typified by higher proportions of *Aerococcus, Atopobium, Bifidobacterium, Corynebacterium, Dialister, Finegoldia, Megasphaera, Mobiluncus, Peptoniphilus, Prevotella, Ralstonia, Staphylococcus, Streptococcus*, and *Sneathia* than other CSTs. The rest of the 13 samples were dominated by the species *Lactobacillus helveticus* or *Lactobacillus delbrueckii*, which were clustered into a new observed community type and named CST VI in this study.

The dominance of CST IV was observed in neonatal samples and postpartum vaginal samples (*n* = 20 and *n* = 17 in groups NO and AD, respectively) (Figure 5B). Most subjects of group BD belonged to CST III and CST VI (15/21) switched to CST IV of group NO. Similarly, they (11/21) shifted to CST IV in the vaginal microbiome after delivery.

## Discussion

In this study, the composition of the vaginal microbiota of women after delivery was characterized and compared with the profile before delivery. Increased microbial richness and diversity were observed in the vaginal samples right after delivery in this study, which was consistent with previous studies with 6 weeks' postpartum samples (13, 27). In addition, dramatic changes in community membership of the vaginal microbiome were observed right after delivery based on PCoA results. In concordance with previous observations (8, 9, 12), *Lactobacillus* was the predominant genus in most of the samples before delivery in this study, along with low proportions of *Atopobium, Gardnerella, Prevotella*, and *Streptococcus*. Whereas, after delivery, *Lactobacillus* significantly decreased, the genera *Prevotella, Staphylococcus, Streptococcus, Thiobacillus, Pseudomonas*, and *Ralstonia* increased significantly. Furthermore, a more diverse ecological network with more nodes and edges was presented in vaginal microbiota after delivery. Taken together, these results demonstrated that the vaginal microbial community right after delivery was altered.

At labor onset, the vaginal microbiome is still dominated by family Lactobacillaceae and Bifidobacteriaceae (28). Moreover, the microbiota of cesarean-born infants partially restore after being exposed to maternal vaginal fluids at birth with sterile gauze and become more and more similar to the microbiota of vaginal delivery infants (29). The previously published results indicate that lactic acid–producing bacteria are still abundant at labor or right after delivery. However, significant declines in abundances of *Lactobacillus* species and an increase in alpha-diversity right after delivery were observed in this study, potentially resulting from the disinfecting operation at labor of the study cohorts. In China, as vaginal examination of standard delivery in the textbook *Obstetrics and Gynecology* (eighth edition) required, soapy water and povidone-iodine are used to clean and disinfect the vulva during delivery in clinic (30). In clinical practice, all gynecologists use cotton ball stained or hand impregnated with povidone-iodine to sterilize the vulva (20). Povidone-iodine is a broad-spectrum antiseptic for preventing skin infections, and its efficacy, particularly on resistant microorganisms, has been shown (31). Its clinical use could alter the vaginal microbial community to a large extent. However, for the deficiency of samples without disinfection in this study, the exact effect of povidone-iodine use on vaginal microbiome was not clear. Follow-up investigation will be taken in our next study.

The vaginal microbiota of women can be generally clustered into five CSTs (9). Four of the CSTs are characterized by high levels of *Lactobacillus crispatus* (CST I), *Lactobacillus gasseri* (CST II), *L. iners* (CST III), and *Lactobacillus jensenii* (CST V). On the other hand, CST IV with reduced *Lactobacillus* species is enriched in a combination of various anaerobic bacteria (9, 32). In this study, the vaginal microbiome was clustered into three major groups by using hierarchical cluster analysis. CST III and CST IV are previously described in North America (33) and European (13, 34), whereas a new CST (named CST VI) dominated by *L. helveticus* was shown in this study. In order to avoid the taxonomic bias caused by the bioinformatics method, the sequences were aligned to the 16S rRNA database in the National Center for Biotechnology Information using BLAST. Meanwhile, the sequence reads were reanalyzed using Mothur software (23). Both of them confirmed that a number of samples were dominated by *L. helveticus*. Previous studies demonstrated that CSTs seem to depend on ethic and biogeographical background (13, 33), and these studies mainly focus on North America and Europe. The new CST dominated by *L. helveticus* might be closely related to our study samples as they all were recruited from Han ethnicity in this study. In further research, large cohort studies should be taken into account to confirm this observation. Beyond this, the V3–V4 regions of 16S rRNA were used to profile the vaginal microbiome community in this study, which was different with a previous study based on *cpn60* gene (32). To cull out the discrepancy caused by the amplified region, lower taxonomic levels should be investigated with whole-genome sequencing in the next study. In addition, the results showed that most samples during pregnancy belonged to CST III and CST VI, with high abundance of *Lactobacillus* species, whereas after delivery the majority of CST III and CST VI before delivery switched to CST IV, in concordance with previous studies (9, 13, 35). Increased rate of CST IV was also observed in neonatal oral samples in this study. CST IV is associated with high Nugent scores and increased risk of bacterial vaginosis, which are linked to preterm birth and chorioamnionitis (13, 36). Further, it has been reported that *Lactobacillus* is the most prevalent and dominant bacterium in female reproductive tract, which appears to ensure normal vaginal microbiota and effectively inhibits the colonization of pathogens through producing lactic acid, H_2_O_2_, and so on (1). Thus, more attention should be paid on the maternal and neonatal health after delivery with *Lactobacillus* decrease induced by using povidone-iodine.

By the time of delivery, the neonate is introduced as the bacteria colonized at the vaginal birth canal (37). Both the PCoA and SourceTracker analysis results consistently indicated that the neonatal oral microbiome was more closely related to the vaginal microbiome after delivery, whereas they were both notably different to the vaginal microbiome before delivery in this study. It indicated that the microbes first colonized at neonatal oral primarily transmitted from the vaginal microbiome right after delivery. That would be helpful to understand the establishment process of the first microbial colonizers of newborns, and it is essential for gaining a comprehensive understanding of neonatal development.

The absence of a longitudinal data set of maternal and neonatal health revealed some insights that need to be determined in further studies. First, during pregnancy, some vaginal microbiota profiles belonged to CST III, some belonged to CST IV, and some were clustered into CST VI. Their long-term impact on maternal and neonatal health should be conducted in the following study. Second, after delivery, some vaginal microbiome communities were still dominated by *Lactobacillus*, whereas others with a small proportion of *Lactobacillus* and similar phenomena were observed in the neonatal oral microbiome. However, the effects of these changes on maternal and neonatal health were not clear. Third, some pregnant women might not need to be disinfected at labor, and some neonates might need to be supplemented with probiotics during the growth and developmental process according to microbial profiles. The clinical operations should be changed according to the result of a large cohort study.

In summary, it was observed in this study that the vaginal microbiome right after delivery was altered, potentially due to perineal disinfection, which affected the colonization of the neonatal oral microbiome. The obvious changes of vaginal microbiome after delivery were the decreased relative abundance of *Lactobacillus* and changed ecological network. These observations will be helpful to understand the community profile of the vaginal microbiome right after delivery in Chinese pregnant women.

## Data Availability Statement

The datasets presented in this study can be found in online repositories. The names of the repository/repositories and accession number(s) can be found below: PRJNA596821.

## Ethics Statement

The studies involving human participants were reviewed and approved by Ethical Committee of Shenzhen Bao'an Maternity and Child Health Hospital. Written informed consent to participate in this study was provided by the participants' legal guardian/next of kin.

## Author Contributions

JY and HL conceived of the presented idea and planned the experiments. JJ and QL carried out the experiments. BX and CN designed the computational framework and analyzed the data. HL, JJ, BX, and JY wrote the manuscript. All authors discussed the results and contributed to the final manuscript.

## Funding

This work was funded by Health and Wellness Commission of Guangxi Zhuang Autonomous Region (20211814), Guangdong Natural Science Fund Project (2018A030313605), Guangzhou Science and Technology Commission Project (201804010090).

## Conflict of Interest

The authors declare that the research was conducted in the absence of any commercial or financial relationships that could be construed as a potential conflict of interest.

## Publisher's Note

All claims expressed in this article are solely those of the authors and do not necessarily represent those of their affiliated organizations, or those of the publisher, the editors and the reviewers. Any product that may be evaluated in this article, or claim that may be made by its manufacturer, is not guaranteed or endorsed by the publisher.
